# Epimedium sagittatum Maxim ameliorates adriamycin‐induced nephropathy by restraining inflammation and apoptosis via the PI3K/AKT signaling pathway

**DOI:** 10.1002/iid3.904

**Published:** 2023-06-14

**Authors:** Ru Wang, Mengnan Zeng, Beibei Zhang, Qinqin Zhang, Shuangshuang Xie, Yingbo Hu, Ruyi Fan, Mengya Wang, Xiao Yu, Yuhan Zhang, Xiaoke Zheng, Weisheng Feng

**Affiliations:** ^1^ School of Pharmacy Henan University of Chinese Medicine Zhengzhou China; ^2^ The Engineering and Technology Center for Chinese Medicine Development of Henan Province Zhengzhou China

**Keywords:** adriamycin‐induced nephropathy, apoptosis, epimedium sagittatum Maxim, inflammation, PI3K/AKT signaling pathway

## Abstract

**Background:**

Modern pharmacological studies show that Epimedium sagittatum Maxim (EPI) has antioxidant, antiapoptotic, anti‐inflammatory effects. However, the effects of EPI on adriamycin‐induced nephropathy are unclear.

**Aim:**

The main purpose of this study is to investigate the effects of EPI on adriamycin‐induced nephropathy in rats.

**Methods:**

The chemical composition of EPI was detected by high performance liquid chromatography. Network pharmacology was used to collect the effects of EPI on adriamycin nephropathy; renal histological changes, podocyte injury, inflammatory factors, oxidative stress levels, apoptosis levels, and the PI3K/AKT signaling pathway were examined. Moreover, analyze the effects of icariin (the representative component of EPI) on adriamycin‐induced apoptosis and PI3K/AKT signaling pathway of NRK‐52e cells.

**Results:**

Network pharmacological results suggested that EPI may ameliorate adriamycin‐induced nephropathy by inhibiting inflammatory response and regulating the PI3K/AKT signaling pathway. The experimental results showed that EPI could improve pathological injury, renal function, podocyte injury, and inhibit inflammation, oxidative stress, apoptosis in adriamycin‐induced nephropathy rats through the PI3K/AKT signaling pathway. Furthermore, icariin inhibited adriamycin‐induced mitochondrial apoptosis in NRK‐52e cells.

**Conclusion:**

This study suggested that EPI ameliorates adriamycin‐induced nephropathy by reducing inflammation and apoptosis through the PI3K/AKT signaling pathway, icariin may be the pharmacodynamic substance basis for this effect.

## Graphical abstract

Epimedium sagittatum Maxim (EPI) can improve podocyte injury, reduce inflammatory response, improve oxidative stress injury and inhibit apoptosis in rats with adriamycin nephropathy, and its mechanism may be related to PI3K/AKT signaling pathway, icariin may be the pharmacodynamic substance responsible for this effect.

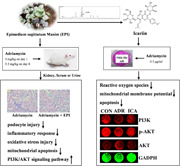

## 1 INTRODUCTION

Adriamycin is an anthracycline antibiotic widely used to treat various cancers.^1^ However, the multiorgan toxicity of adriamycin (renal toxicity) limits its clinical application.^2^ Adriamycin induced nephropathy model is similar to human nephropathy, characterized by abnormal thickening of glomerular basement membrane, glomerular sclerosis, and tubule interstitial inflammation.^3^, ^4^ Previous studies have shown that adriamycin injected into rodents rapidly distributes into kidney tissue and causes kidney damage, including podocyte damage, severe inflammation, and glomerulosclerosis.^5^, ^6^ In addition, the quinone structure of adriamycin can produce reactive oxygen species to induce peroxidation, destroy the integrity of the glomerular filtration membrane, and even lead to proteinuria.^7^, ^8^ Currently, glucocorticoids are the main treatment for adriamycin‐induced nephropathy; however, glucocorticoid treatment is frequently has serious side effects, and patients are prone to developing drug resistance.^9^ Therefore, it is of substantial clinical significance to investigate drugs for adriamycin‐induced nephropathy with good efficacy and few side effects.

Epimedium sagittatum Maxim (EPI), a berberis herb flower, enjoys a strong reputation in traditional Chinese medicine and has been diffusely used in Asian countries since ancient times due to its various effective chemical components.^10^ Modern pharmacology has shown that EPI has anti‐inflammatory, antioxidant, antiapoptosis, and antitumor effects.^11^, ^12^ Studies have shown that active components of EPI flavonoids can improve renal function impairment in chronic kidney disease and alleviate chronic renal failure by exerting anti‐inflammatory and antioxidant effects.^13^, ^14^ However, the ameliorative effects of EPI on adriamycin‐induced nephropathy and its related mechanisms have not been reported. Therefore, this study explored the improvement effect of EPI on rats with nephropathy induced via tail‐vein injection of adriamycin, providing theoretical basis and new ideas for clinical treatment adriamycin nephropathy.

## 2 MATERIALS AND METHODS

### 2.1 Plant material and reagents

The above‐ground parts of EPI were gathered from the Epimedium planting base in Fenghui County, Zhumadian, Henan Province in September 2020. One copy of the certificate (No.: 20200960) was stored in the School of Pharmacy, Henan University of Chinese Medicine. Adriamycin (H44024359) was purchased from Main Luck. Prednisone acetate (P829896) was purchased from Macklin. Icariin (S18010) was purchased from Solarbio.

### 2.2 High performance liquid chromatography (HPLC) analysis

Shimadzu LC‐40 HPLC system was used for chromatographic analysis of EPI and icariin. Reversed phase C18 ODS column (10ID × 250 mm, Cosmosil 5C18‐MS‐II Packed column, Nacalai Tesque) was used for the column. Column temperature 30°C, The flow rate was set to 2.0 mL/min and the detection wavelength was 210 nm. The mobile phase was 0.1% phosphoric acid solution (A)‐acetonitrile (B), the linear elution procedure was 0–50 min, 10%–100% B.

### 2.3 Data collection

The EPI targets were acquired from TCMSP (https://old.tcmsp-e.com/tcmsp.php). The selected target protein names were annotated based on the UniProt database (https://www.UniProt.org/). The genes related with adriamycin nephropathy were gained from GeneCards (https://www.genecards.org/) and OMIM (https://www.omim.org), jvenn (http://jvenn.toulouse.inra.fr/app/example.html) was used to find EPI and adriamycin nephropathy common target genes. The interaction network between proteins was investigated using the STRING database (https://cn.string-db.org/) and used to explore the core targets of EPI in adriamycin‐induced nephropathy. KEGG pathway analyses were analyzed by bioinformatics (www.bioinformatics.com.cn).

### 2.4 Animals

**Figure 1:**
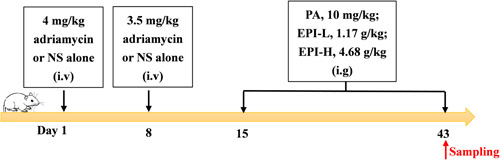
Timeline of the adriamycin‐induced nephropathy model. Male SD rats were given 4 mg/kg adriamycin or saline via the tail vein on day 1 and 3.5 mg/kg on day 8. PA, EPI, or distilled water were administered from day 15, and samples were collected after 4 consecutive weeks. NS: normal saline; i.v: tail vein injection; i.g: intragastric administration. EPI, Epimedium sagittatum Maxim; PA, prednisone acetate; SD, Sprague Dawley.

A total of 60 male Sprague‐Dawley rats (6–8 weeks, specific pathogen free, 180–220 g) were purchased from Ji Nan Peng Yue Experimental Animal Breeding Co., LTD. (License: SCXK [LU] 20190003). Animals housed in a cleaner‐level room with a 12‐h light/dark cycle and a constant temperature of 24°C, with free access to food and water. After 7 days of adaptive feeding, 10 rats were randomly selected for the control group (CON, *n* = 10, group 1) and the remaining rats received adriamycin injection. The adriamycin‐induced nephropathy (ADR) rats were first injected with adriamycin diluted with 0.9% normal saline at a dose of 4 mg/kg via the tail vein on day 1 and were given a second dose of adriamycin at a dose of 3.5 mg/kg via the tail vein on day 8. Normal‐group rats were injected with the same dose of normal saline in the tail vein. On day 15, ADR rats were randomly divided into the ADR (ADR, *n* = 14, group 2), prednisone acetate (PA, 10 mg/kg, *n* = 12, group 3), Epimedium sagittatum maxim low‐dose (EPI‐L, 1.17 g/kg, *n* = 12, group 4), and Epimedium sagittatum maxim high‐dose (EPI‐H, 4.68 g/kg, *n* = 12, group 5) groups. The PA, EPI‐L, and EPI‐H groups were intragastrically administered the corresponding drugs daily for 4 weeks; those in the CON and ADR groups received distilled water only. All rats were euthanized by excessive anesthesia (intraperitoneal injection of 100 mg/kg ketamine + 10 mg/kg xylazine), and abdominal aorta blood was collected and analyzed. The timeline of the adriamycin‐induced nephropathy model is shown in Figure 1.

### 2.5 Histological examination

The upper part of the left kidney of rats, three in each group, was soaked in 4% paraformaldehyde fixative solution for 24 h, dehydrated with alcohol gradient, paraffin was used for embedding, cut into 5 μm sections by microslicer, and stained with hematoxylin and eosin (H&E). The severity of kidney histopathology was scored on the basis of interstitial inflammatory cell infiltration, glomerular basement membrane thickening, and glomerular vacuolar degeneration by Yixiao Xu et al.^15^, ^16^

### 2.6 Transmission electron microscopy

The lower part of the left kidney of rats with the size of 1 × 1 × 1 mm^3^ was selected, three in each group, and soaked in osmium (1%) overnight, followed by incubation in 0.1 M phosphoric acid buffer for 2 h at 20°C. After dehydration, infiltration, and slices were taken 60–80 nm thick and stained with the uranium lead double staining method. Ultra‐thin sections were observed with an electron microscope (HT7700, Hitachi).

### 2.7 Enzyme‐linked immunosorbent assay (ELISA) analysis

After intragastric administration, the rats were placed in metabolic cages with eight rats in each group, and urine was collected for 8 h. The rats were anesthetized and blood was taken from the abdominal aorta. The collected urine and blood were centrifuged at 3000 rpm for 15 min to acquire the supernatant, which was stored at 4°C for later use. The levels of microscale albuminuria (MAU, E‐EL‐R0025c, Elabscience) in the urine and kidney injury molecule‐1 (KIM‐1, E‐EL‐R3019, Elabscience), interleukin‐10 (IL‐10, KE20003, Proteintech), and transforming growth factor‐β1 (TGF‐β1, KE20010, Proteintech) in the serum were measured using an ELISA kit according to directions.

### 2.8 Assessments of biochemical parameters

Eight rats in each group were collected in a metabolic cage for urine collection, then blood was collected through the abdominal aorta, and supernatant was obtained by centrifugation at 3000 rpm for 15 min. The right kidney of these eight rats was extracted to obtain kidney tissue homogenate according to the kit instructions. The levels of creatinine (Cr, C011‐2‐1, Nanjing Jiancheng), urea nitrogen (BUN, C013‐2‐1, Nanjing Jiancheng), urinary protein (UP, C035‐2‐1, Nanjing Jiancheng) in the serum and urine, superoxide dismutase (T‐SOD, A001‐1, Nanjing Jiancheng), glutathione peroxidase (GSH‐Px, A005, Nanjing Jiancheng) and micro malondialdehyde (MDA, A003‐1, Nanjing Jiancheng) in the kidney tissue were measured.

### 2.9 Real‐time quantitative PCR (RT‐qPCR) analysis

The upper part of the left kidney was selected, with three rats in each group, total RNA was extracted using total RNA extraction kit (R1200, Solarbio). Quantification was performed by NanoDrop™ One ultraviolet spectrophotometer (Thermo Scientific), followed by reverse transcription using BeyoRTTM III First Strand cDNA Synthesis Kit (D7178M, Beyotime) to obtain template DNA. Finally, the levels of tumor necrosis factor‐α (TNF‐α), interleukin‐6 (IL‐6), interleukin‐1β (IL‐1β), monocyte chemoattractant protein‐1 (MCP‐1), nephrin, desmin, phosphoinositide 3‐Kinase (PI3K), and serine/threonine‐protein kinase (AKT) were detected using QuantiNova™ SYBR green PCR kit (Qiagen). Table 1 shows the sequence of primers used for RT‐qPCR amplification.

**Table 1:** The primers sequence of RT‐qPCR.

Gene	Primer sequences (5′–3′)
*Rat TNF‐α*	F: CCAGGTTCTCTTCAAGGGACAA
	R: GGTATGAAATGGCAAATCGGCT
*Rat IL‐6*	F: AGGATACCACCCACAACAGACC
	R: AGGATACCACCCACAACAGACC
*Rat IL‐1β*	F: TGACCTGTTCTTTGAGGCTGAC
	R: CATCATCCCACGAGTCACAGAG
*Rat MCP‐1*	F: TGCAGGTCTCTGTCACGCTTC
	R: AGTGAATGAGTAGCAGCAGGTGA
*Rat Nephrin*	F: GTCGGAGGACAGGATCAGGAAT
	R: TCAGGCCAGCGAAGGTCATAG
*Rat Desmin*	F: CAGGAACAGCAGGTCCAGGTAG
	R: GAGTCGTTGGTGCCCTTGAGA
*Rat PI3K*	F: CCTGGTGATTGAGAAGTGTAAAGTG
	R: CGTAAGGCAGAAGGCACAGGT
*Rat AKT*	F: CGACGTAGCCATTGTGAAGGAG
	R: ATTGTGCCACTGAGAAGTTGTTG
*Rat GAPDH*	F: CTGGAGAAACCTGCCAAGTATG
	R: GGTGGAAGAATGGGAGTTGCT

Abbreviations: GAPDH, glyceraldehyde 3‐phosphate dehydrogenase; IL, interleukin; MCP‐1, monocyte chemoattractant protein‐1; TNF, tumor necrosis factor.

### 2.10 Flow cytometry analysis

Three rats in each group were selected from the lower part of the left kidney, then fresh kidney tissue was cut into pieces and washed twice with cold phosphate buffer saline (PBS), and digested with pancreatic enzymes for 2 min. Stop digestion with fetal bovine serum (FBS), and 70 μm membrane filter was used to obtain the renal primary cells. The Reactive Oxygen Species Assay Kit (CA1410, Solarbio) was used to detect the levels of reactive oxygen species (ROS), Fluo‐4, an AM fluorescent probe (F8500, Solarbio) was used to measure the levels of Ca^2+^, the JC‐1 Mitochondrial Membrane Potential Detection Kit (G1511, Servicebio) was used to measure the levels of mitochondrial membrane potential (MMP), and the apoptosis kit (G1515, Servicebio) was used to measure apoptosis levels. Before staining macrophages and neutrophils, Fc‐γR was blocked with anti‐CD16/32 Ab (553142, BD) for 15 min, and stained with the following combination of antibodies for 30 min on ice: F4/80‐Alexa Fluor (8045596, BD Biosciences), CD11b‐APC (2311195, Invitrogen), Ly6G‐FITC (2440387, Invitrogen), and CD11b‐FITC (2172513, Invitrogen).

### 2.11 Immunohistochemistry

The upper part of the left kidney was taken from rats and soaked in 4% paraformaldehyde fixative for 24 h, then embedded with paraffin, dewaxed, dehydrated and cut into 5 mm thick segments. The tissue segments were incubated with anti‐Nephrin (1:100) and anti‐desmin (1:100), and followed by Goat Anti‐Rabbit IgG H&L (horseradish peroxidase) (1:300). Hematoxylin was used as a counterstain and neutral balsam was used to mount the sections for observation. Under the electron microscope, the tan area showed positive expression.

### 2.12 Western blotting analysis

The upper part of the left kidney was selected from three rats, total tissue protein was extracted from kidney tissue by using total protein extraction kit (BC3710, Solarbio), and bicinchoninic acid assay protein quantification kit (PC0020, Solarbio) was used to determine its concentration. The sample size was 60 μg for sodium dodecyl sulfate–polyacrylamide gel electrophoresis. Subsequently, the membrane was incubated in 5% bovine serum albumin (BSA) for 1.5 h at room temperature. Then the primary antibodies were added: nuclear factor kappa B (NF‐κB p65, ab16502, Abcam), phosphorylated NF‐κB (NF‐κB p‐p65, ab86299, Abcam), IL‐1β (ab9722, Abcam), B‐cell lymphoma‐2 (Bcl‐2, ab59348, Abcam), BCL2‐Associated X (Bax, ab32503, Abcam), Cleaved‐caspase‐3 (ab2302, Abcam), Caspase‐3 (ab13847, Abcam), Nuclear factor erythroid2‐related factor 2 (Nrf2, ab137550, Abcam), Phospho‐Nrf2 (p‐Nrf2, ab76026, Abcam), Heme Oxygenase‐1 (HO‐1, A19062, ABclonal), NAD(P)H Quinone Dehydrogenase 1 (NOQ‐1, A19586, ABclonal), PI3K (ab1678, Abcam), AKT (ab8805, Abcam), Phospho‐Akt (p‐AKT, ab66138, Abcam) and β‐actin (Ac026, ABclonal). The membranes were incubated with the antibodies for 3 h. Subsequently, secondary antibody (goat anti‐rabbit 925‐68071, Li‐COR) was added and the membranes were incubated in the dark for 1 h. The levels of protein expression were quantified by using Studio version 5.2 (Clx, Li‐COR).

### 2.13 Cell culture

Normal rat renal epithelial cell line NRK‐52e was purchased from American Type Culture Collection, added to Dulbecco's modified Eagle medium medium (100 IU/mL penicillin and 100 mg/mL streptomycin) supplemented with 10% FBS and incubated at 37°C in a 5% CO_2_ incubator. Cells in logarithmic growth phase were used for the experiments.

### 2.14 Cell viability assay

NRK‐52e cells in 96‐well plates at a density of 4000 cells/well and divided into the control (CON) and adriamycin (0.25, 0.5, 1, 2, and 5 μg/mL) groups; after 24 h, a methyl thiazolyl tetrazolium (MTT) assay was conducted to determine cell viability. Then, NRK‐52e cells in a 96‐well plate at a density of 4000 cells/well and divided into the control (CON), adriamycin (0.5 μg/mL), and icariin (0.5, 1, 5, 10, 20, and 50 μM + 0.5 μg/mL adriamycin) groups. Cell viability was determined by MTT assay after 24 h culture. All experiments were repeated three times.

### 2.15 Flow cytometry analysis of NRK‐52e cells

NRK‐52e cells in 6‐well plate at a density of 120,000 cells/well. Overnight incubation, cells were divided into control (CON), adriamycin (0.5 μg/mL), and icariin (10 μM + 0.5 μg/mL adriamycin) groups. After 24 h, the Reactive Oxygen Species Assay, JC‐1 Mitochondrial Membrane Potential Detection, and apoptosis kits were used to measure the levels of ROS, MMP, and apoptosis according to the kit instructions.

### 2.16 In‐cell western blot analysis

NRK‐52e cells in 96‐well plates at a density of 4000 cells/well. 24 h after treatment as described above, the cells were fixed in 3.7% paraformaldehyde for 20 min and 5 permeations with 0.1% Triton X‐100 for 5 min each. The cells were blocked with 5% BSA for 1.5 h and PI3K, AKT, p‐AKT, and glyceraldehyde 3‐phosphate dehydrogenase (AC033, ABclonal) antibodies were added and incubated overnight at 4°C. After PBS (PBST) washing, the cells were incubated with goat Anti‐Rabbit IgG (925‐68071, LI‐COR) or Goat anti‐mouse IgG (925‐32210, LI‐COR) and incubated for 1 h away from light and quantification using Image Studio version 5.2.

### 2.17 Statistical analysis

Data were analyzed by SPSS 26.0 software. Results were expressed as mean ± standard deviation. One‐way analysis of variance was used for comparison between groups. *p* < .05 indicates a significant difference and *p* < .01 indicates a highly significant difference in results.

## 3 RESULTS

### 3.1 HPLC analysis of EPI

**Figure 2:**
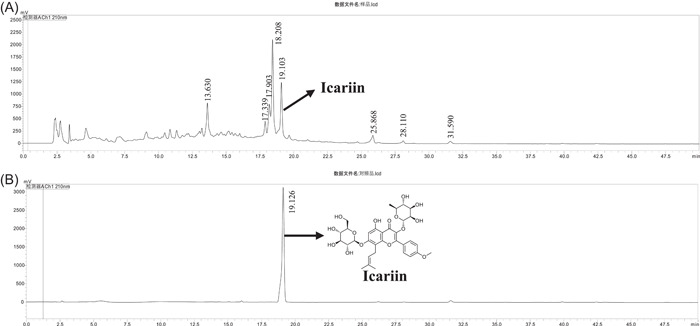
HPLC analysis of (A) EPI and (B) icariin. EPI, Epimedium sagittatum Maxim; HPLC, high performance liquid chromatography.

The chromatogram of EPI by HPLC is shown in Figure 2. EPI contains a variety of chemical components and a variety of small molecular compounds. By comparison with the standard product of icariin, we detected the peak of icariin from EPI.

### 3.2 Effects of EPI on histopathology and functions of kidney in adriamycin‐induced nephropathy rats

**Figure 3:**
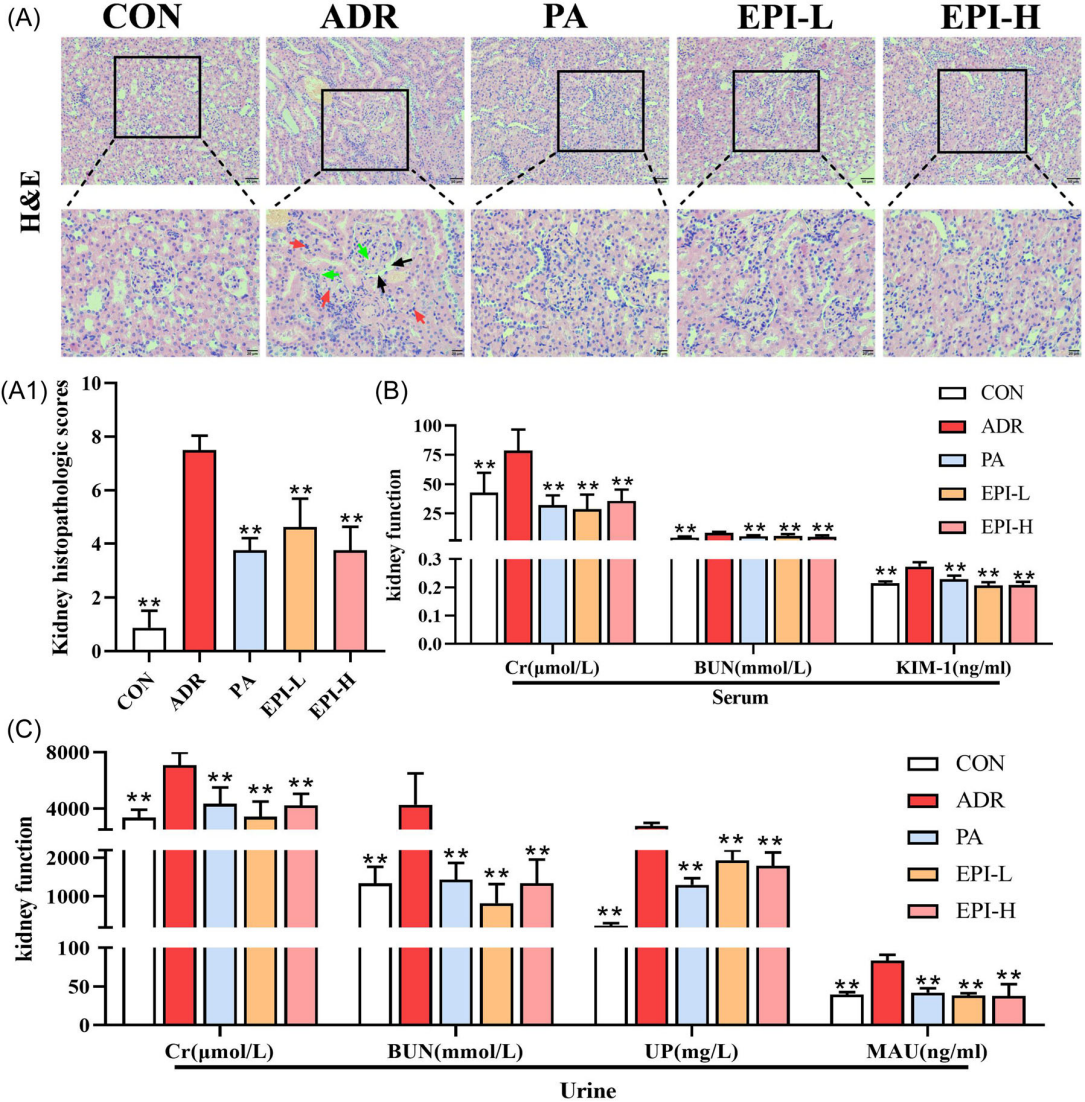
Effects of EPI on histopathology and functions of kidney in adriamycin‐induced nephropathy rats. CON: control. (A) Kidney tissue stained with H&E (×200 and ×400). Red arrows—severe infiltration of inflammatory cells, black arrows—thickening of the glomerular basement membrane, and green arrows—vacuolar degeneration of tubular epithelial cells. (B) Effects of EPI on serum levels of Cr, BUN, and KIM‐1 in rats with nephropathy. (C) Effects of EPI on urine levels of Cr, BUN, MAU, and UP in adriamycin‐induced nephropathy rats. Data are expressed as the mean ± standard deviation (*SD*), *n* = 3 or 8 rats per group, *p < .05, **p < .01 compared with the ADR group. ADR, adriamycin‐induced nephropathy; EPI‐H, Epimedium sagittatum Maxim high dose group; EPI‐L, Epimedium sagittatum Maxim low dose; H&E, hematoxylin and eosin; PA, prednisone.

H&E staining of the kidney revealed severe interstitial inflammatory cell infiltration, blurred glomerular basement membrane boundaries, hyperplasia, and vacuolar degeneration of kidney tubular epithelial cells. PA and EPI improved adriamycin‐induced inflammatory cell infiltration, glomerular basement membrane thickening, and epithelial vacuolar degeneration (Figure 3A). In addition, the levels of Cr, BUN, and KIM‐1 in the serum and the levels of Cr, BUN, MAU, and UP in the urine were significantly increased in the ADR group; these changes were effectively reversed by PA and EPI (Figure 3B,C).

### 3.3 Effects of EPI on podocyte injury in adriamycin‐induced nephropathy rats

**Figure 4:**
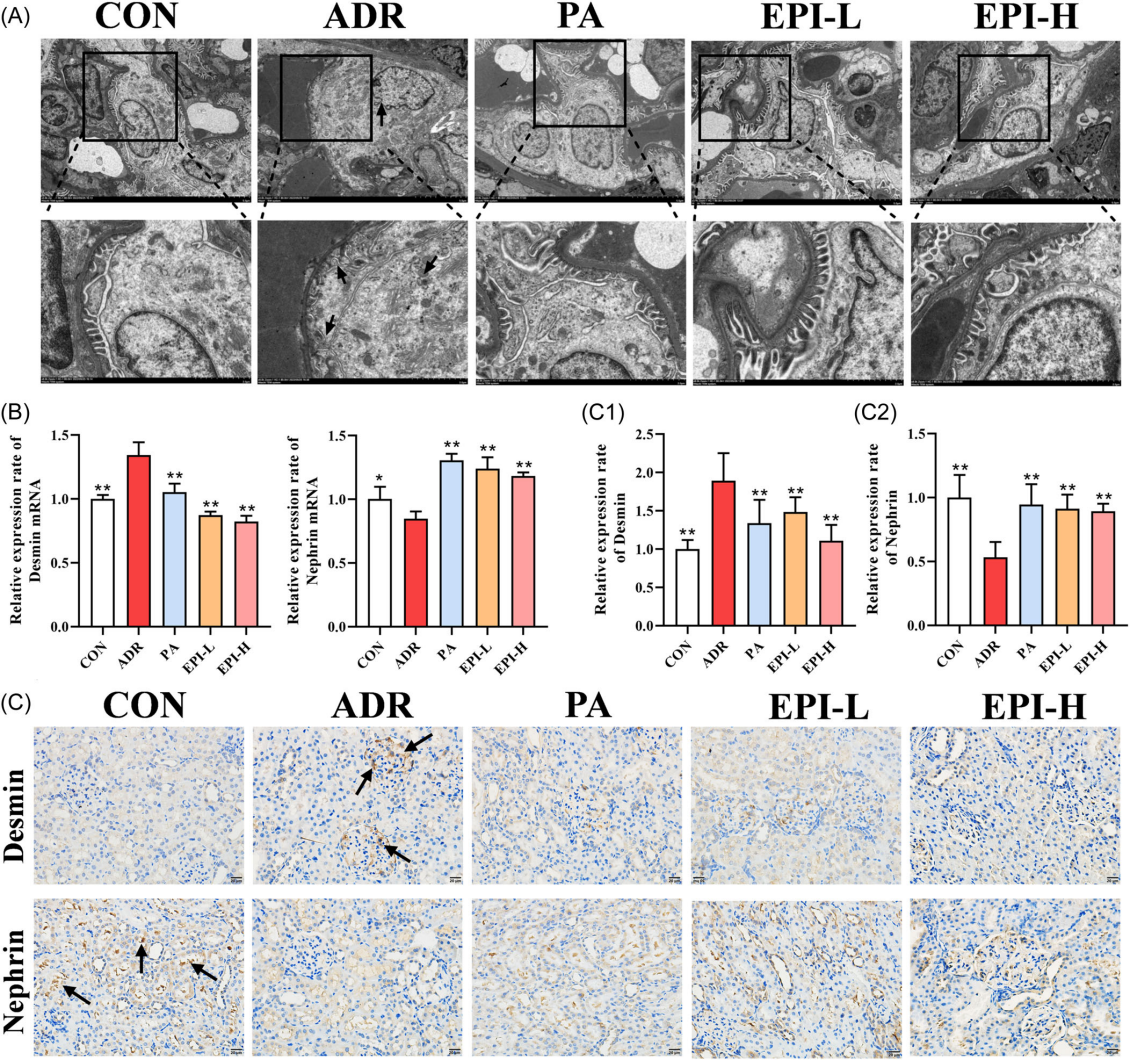
Effects of EPI on podocyte injury in adriamycin‐induced nephropathy rats. CON: control. (A) Kidney tissue transmission electron microscopy (×3000 and ×8000). The black arrow shows the fusion of the foot process of the renal tissue and the thickening of the glomerular basement membrane. (B) The relative mRNA expression levels of nephrin and desmin in kidney tissue were measured by RT‐qPCR. (C) The protein expression levels of nephrin and desmin were measured by immunohistochemistry. The black arrows in the figure indicate that tan is a positive expression, and the darker the color, the higher the protein expression. Data are expressed as the mean ± *SD*, *n* = 3 rats per group, *p < .05, **p < .01 compared with the ADR group. ADR, adriamycin‐induced nephropathy; EPI‐H, Epimedium sagittatum Maxim high dose group; EPI‐L, Epimedium sagittatum Maxim low dose; H&E, hematoxylin and eosin; mRNA, messenger RNA; PA, prednisone.

The ultrastructure of the kidney tissue showed extensive loss of foot process fusion and thickening of the glomerular basement membrane in rats with adriamycin‐induced nephropathy. However, these changes were attenuated by PA and EPI (Figure 4A). Moreover, in adriamycin‐induced nephropathy rats, the relative messenger RNA (mRNA) expression level of nephrin was significantly decreased and that of desmin was increased (Figure 4B); the protein expression level of nephrin was significantly reduced, and that of desmin was increased (Figure 4C). All of these effects were reversed by PA and EPI.

### 3.4 Effects of EPI on adriamycin‐induced nephropathy based on network pharmacology

**Figure 5:**
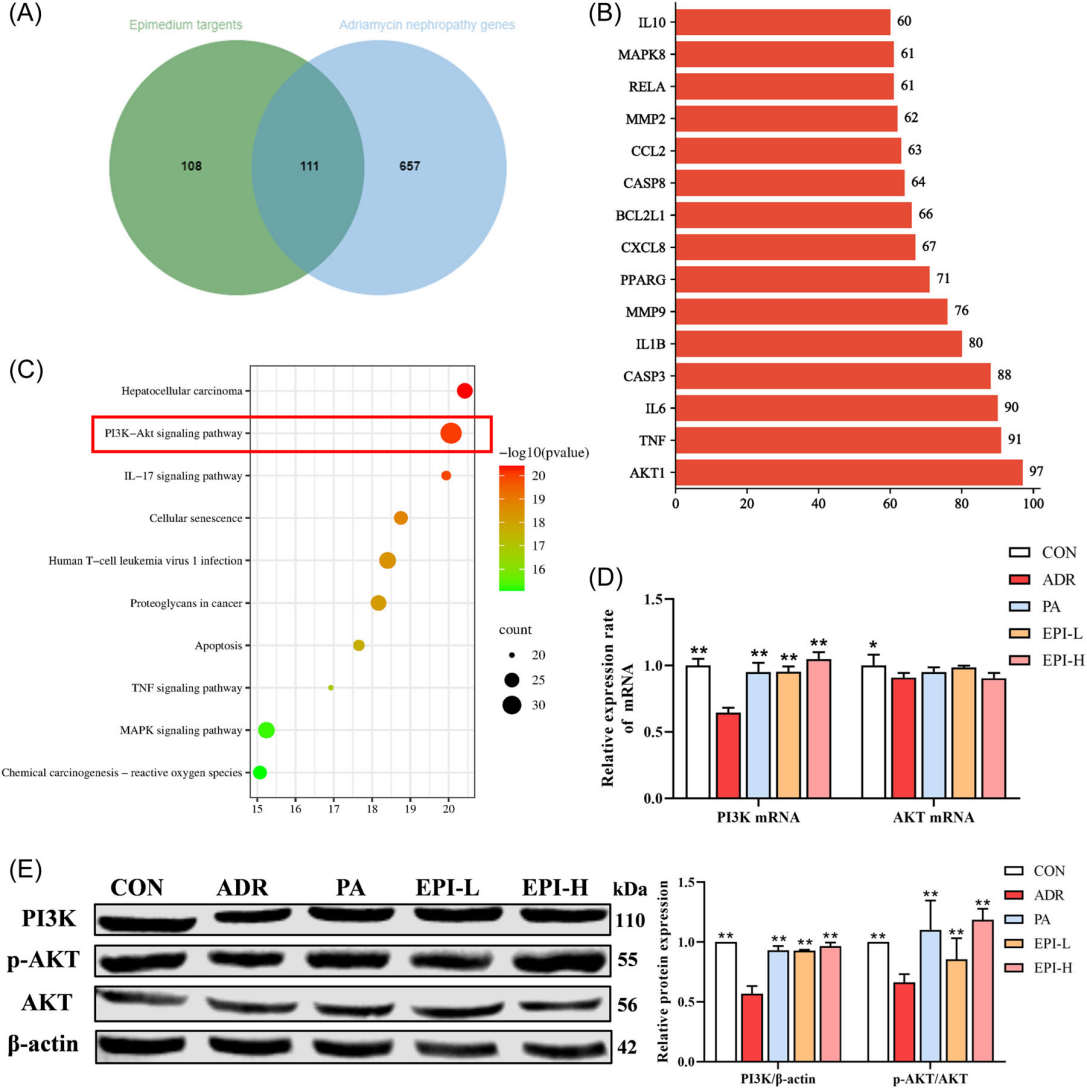
Effects of EPI on adriamycin‐induced nephropathy based on network pharmacology. CON: control. (A) The target genes of EPI in adriamycin‐induced nephropathy were identified by jvenn. (B) Core gene histogram of EPI for adriamycin nephropathy. (C) KEGG enrichment analysis of EPI in adriamycin nephropathy. (D) The relative expression levels of PI3K and AKT mRNA in kidney tissue were measured by RT‐qPCR. (E) Western blot detection of PI3K/AKT signaling pathway‐related protein levels. Data are expressed as the mean ± *SD*, *n* = 3 rats per group, *p < .05, **p < .01 compared with the ADR group. ADR, adriamycin‐induced nephropathy; EPI‐H, Epimedium sagittatum Maxim high dose group; EPI‐L, Epimedium sagittatum Maxim low dose; H&E, hematoxylin and eosin; mRNA, messenger RNA; PA, prednisone.

A total of 219 EPI related target genes were obtained from TCMSP (oral bioavailability ≥30%, drug similarity ≥0.18), and 768 genes related to adriamycin nephropathy were obtained from GeneCards and OMIM. Furthermore, 111 target genes of EPI in the treatment of adriamycin nephropathy were acquired by jvenn (Figure 5A), and protein interaction network among targets was constructed by importing data into STRING, the core genes included AKT1, TNF, and IL6 (Figure 5B). KEGG signal path analysis results are shown in Figure 5C, indicating that PI3K/AKT signaling pathway was most predominantly associated with EPI in the treatment of ADR. The PI3K/AKT signaling pathway can reduce cellular oxidative stress, inhibit the inflammatory response, and improve podocyte injury; the pathogenesis of adriamycin‐induced nephropathy involves functions related to this pathway.^9^, ^17^ To verify the predicted results of network pharmacology and to find the signaling pathway related to the EPI treatment of adriamycin‐induced nephropathy, we first measured the levels of protein and mRNA associated with the PI3K/AKT pathway, and the results showed that EPI could significantly increase the levels of PI3K and p‐AKT in adriamycin‐induced nephropathy rats (Figure 5D,E).

### 3.5 Effects of EPI on the inflammatory response in adriamycin‐induced nephropathy rats

**Figure 6:**
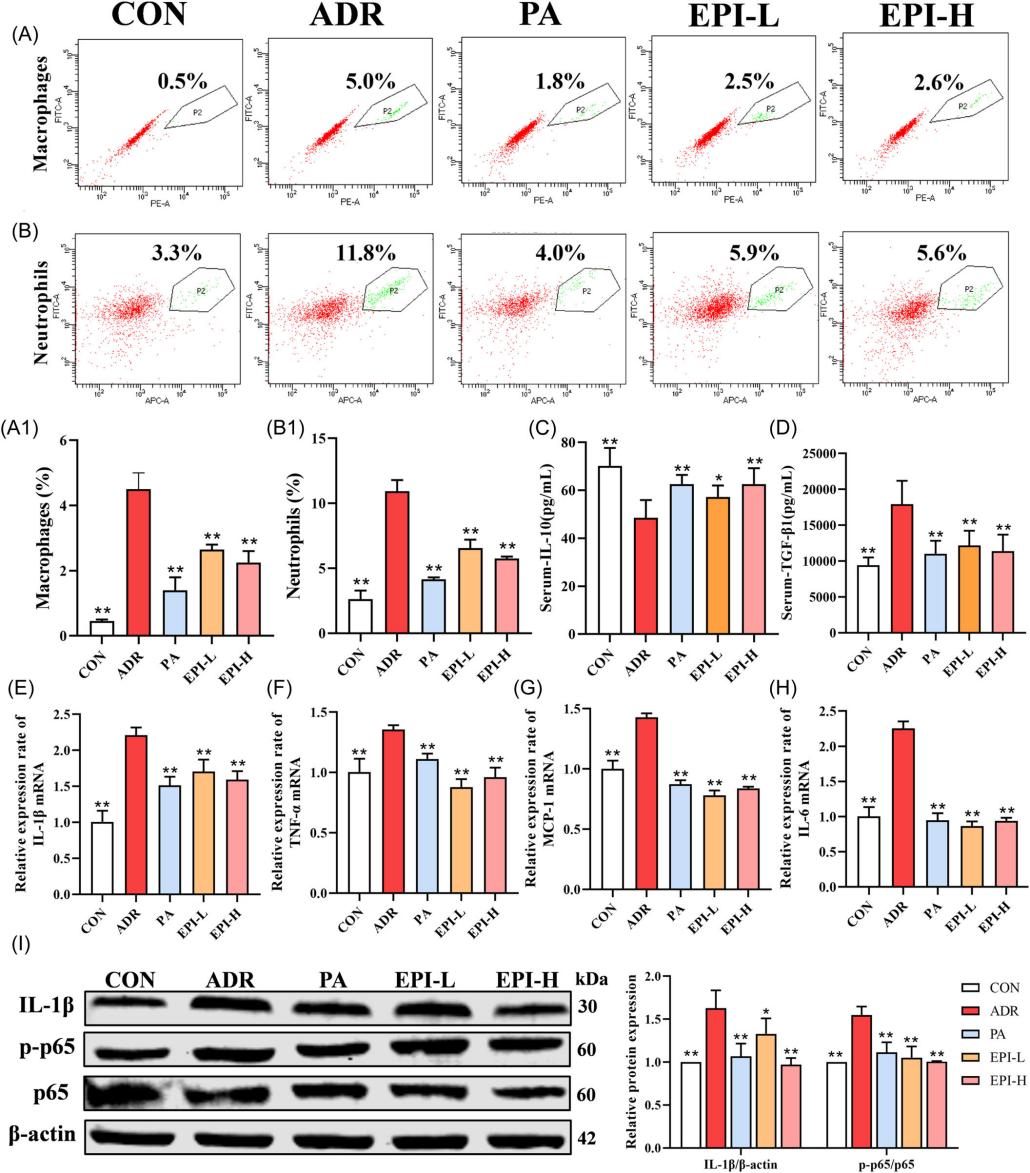
Effects of EPI on the inflammatory response of adriamycin‐induced nephropathy rats. CON: control. (A) The level of macrophages in kidney tissue was measured by flow cytometry. (B) The level of neutrophils in kidney tissue was measured by flow cytometry. (C, D) The levels of IL‐10 and TGF‐β1 in the serum were measured by ELISA. (E–H) The levels of IL‐1β, TNF‐α, MCP‐1, and IL‐6 in kidney tissue were measured by RT‐qPCR. (I) The NF‐κB signaling pathway‐related proteins were measured by western blot analysis. Data are expressed as the mean ± *SD*, *n* = 3 or 8 rats per group, *p < .05, **p < .01 compared with the ADR group. ADR, adriamycin‐induced nephropathy; EPI‐H, Epimedium sagittatum Maxim high dose group; EPI‐L, Epimedium sagittatum Maxim low dose; H&E, hematoxylin and eosin; mRNA, messenger RNA; PA, prednisone; TGF, transforming growth factor; TNF, tumor necrosis factor.

As shown in Figure 6A,B, the levels of macrophages and neutrophils in primary renal cells significantly increased in adriamycin‐induced nephropathy rats. In addition, the level of TGF‐β1 was significantly increased, and that of IL‐10 was decreased, in the serum (Figure 6C,D). Relative expression of IL‐6, MCP‐1, IL‐1β, and TNF‐α mRNA were significantly increased in kidney tissue (Figure 6E–H), AND the levels of IL‐1β and p‐p65 were increased in adriamycin‐induced nephropathy rats (Figure 6I). All of these effects were attenuated by PA and EPI.

### 3.6 Effects of EPI on oxidative stress and the mitochondrial apoptosis pathway in adriamycin‐induced nephropathy rats

**Figure 7:**
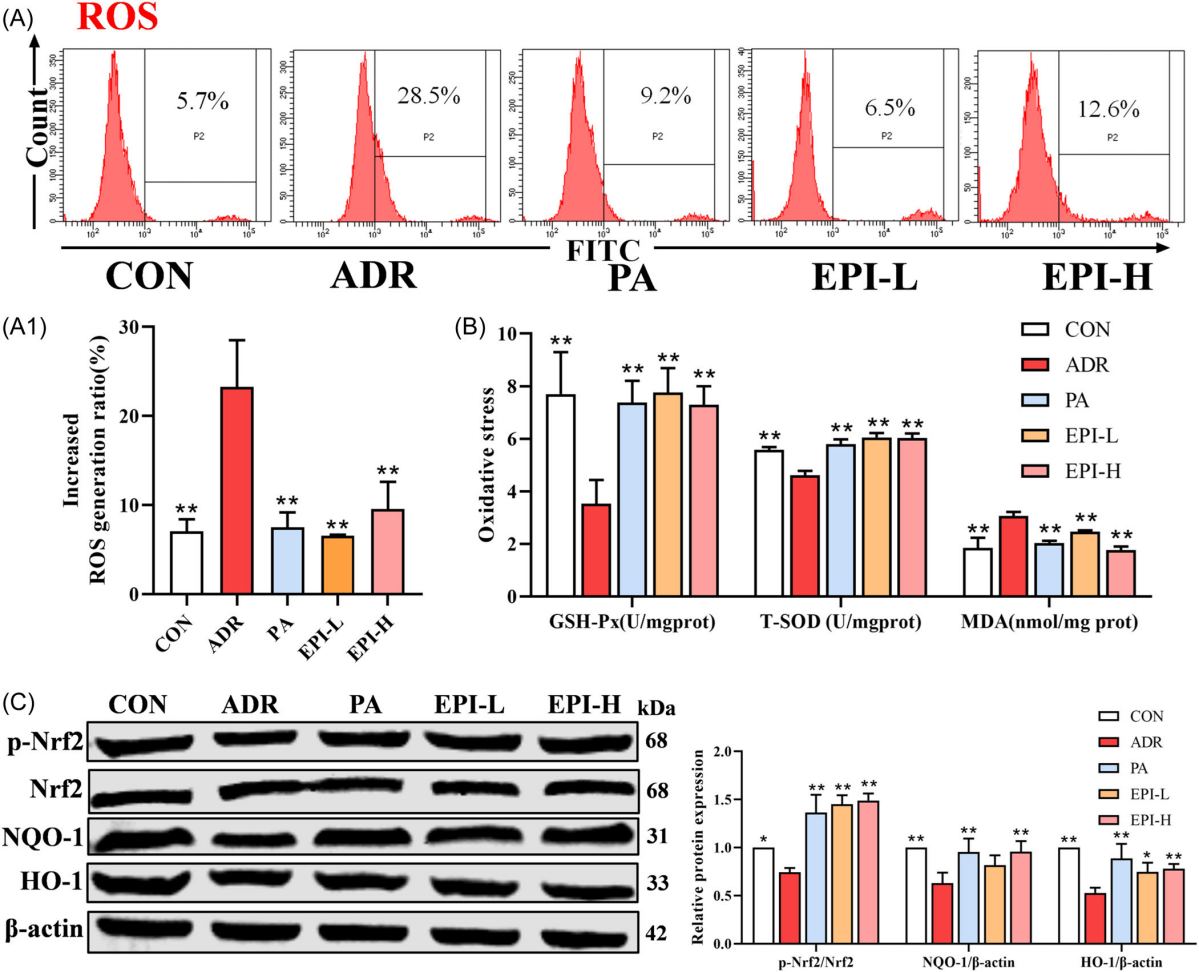
Effects of EPI on oxidative stress in adriamycin‐induced nephropathy rats. CON: control. (A) The levels of ROS in primary renal cells were measured by flow cytometry. (B) The oxidative stress‐related markers were measured by the cuvette assay. (C) The oxidative stress signaling pathway‐related proteins were measured by Western blot analysis. Data are expressed as the mean ± *SD*, *n* = 3 or 8 rats per group, *p < .05, **p < .01 compared with the ADR group. ADR, adriamycin‐induced nephropathy; EPI‐H, Epimedium sagittatum Maxim high dose group; EPI‐L, Epimedium sagittatum Maxim low dose; ROS, reactive oxygen species.

**Figure 8:**
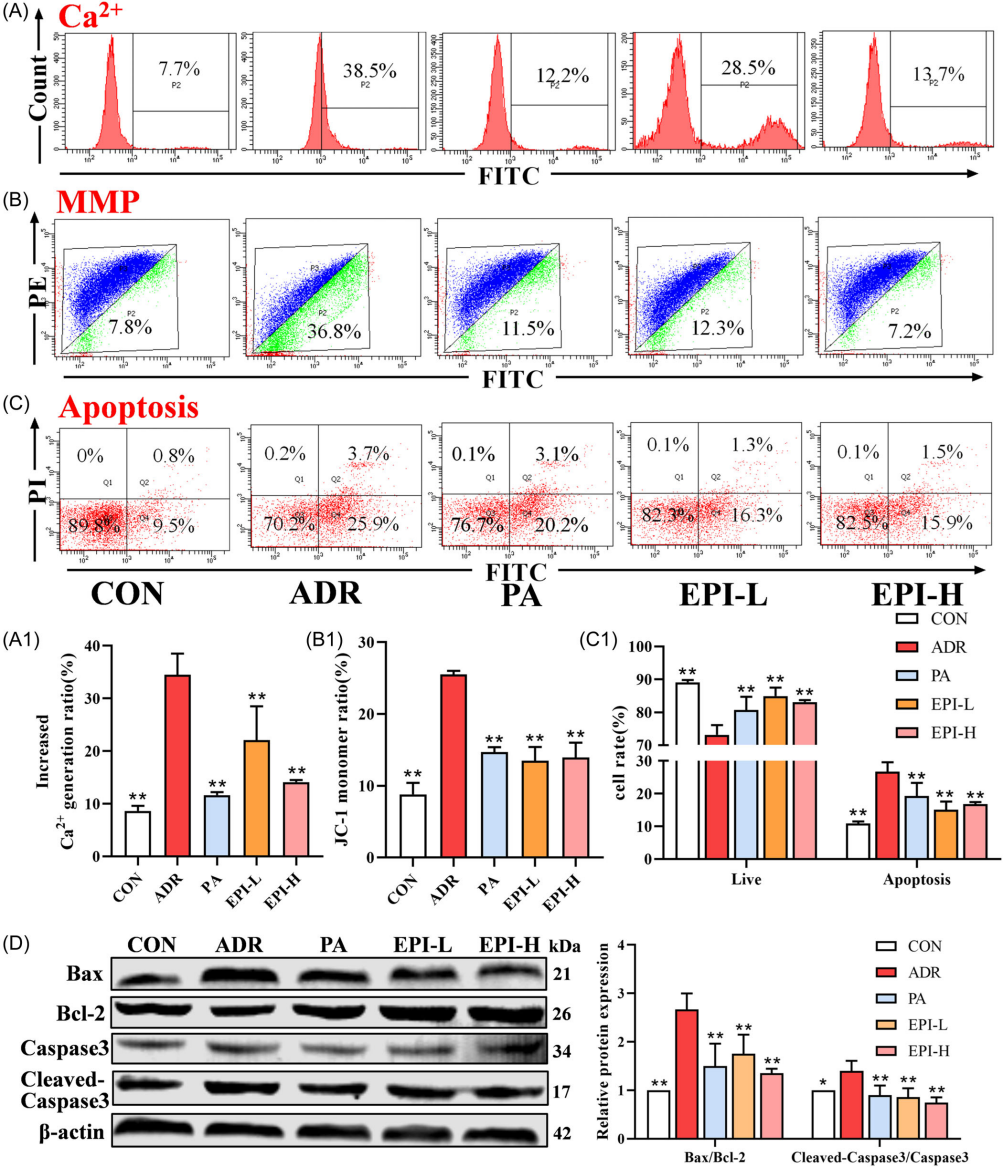
Effects of EPI on the mitochondrial apoptosis pathway in adriamycin‐induced nephropathy rats. CON: control. (A) The levels of Ca^2+^ in primary renal cells were measured by flow cytometry. (B) The levels of MMP in primary renal cells were measured by flow cytometry. (C) The level of apoptosis in primary renal cells was measured by flow cytometry. (D) The apoptosis signaling pathway‐related proteins were measured by Western blot analysis. Data are expressed as the mean ± *SD*, *n* = 3 rats per group, *p < .05, **p < .01 compared with the ADR group. ADR, adriamycin‐induced nephropathy; EPI‐H, Epimedium sagittatum Maxim high dose group; EPI‐L, Epimedium sagittatum Maxim low dose; MMP, mitochondrial membrane potential.

As shown in Figure 7A,B, the levels of GSH‐Px and T‐SOD were decreased and MDA and ROS were increased in adriamycin‐induced nephropathy rats, which could be reversed by PA and EPI. In addition, the protein levels of p‐Nrf2, HO‐1, and NQO‐1 were upregulated after treatment with PA and EPI (Figure 7C). The levels of Ca^2+^ and apoptosis were also significantly increased, and MMP levels were decreased, in adriamycin‐induced nephropathy rats; these effects could be significantly reversed by PA and EPI (Figure 8A–C). The expression levels of Cleaved‐Caspase3 and Bax were significantly increased, and Bcl‐2 levels were decreased, in adriamycin‐induced nephropathy rats, while PA and EPI could decrease the levels of Cleaved‐Caspase3 and Bax and increase the levels of Bcl‐2 (Figure 8D).

### 3.7 Effects of icariin on mitochondrial apoptosis in adriamycin‐induced NRK‐52e cells

**Figure 9:**
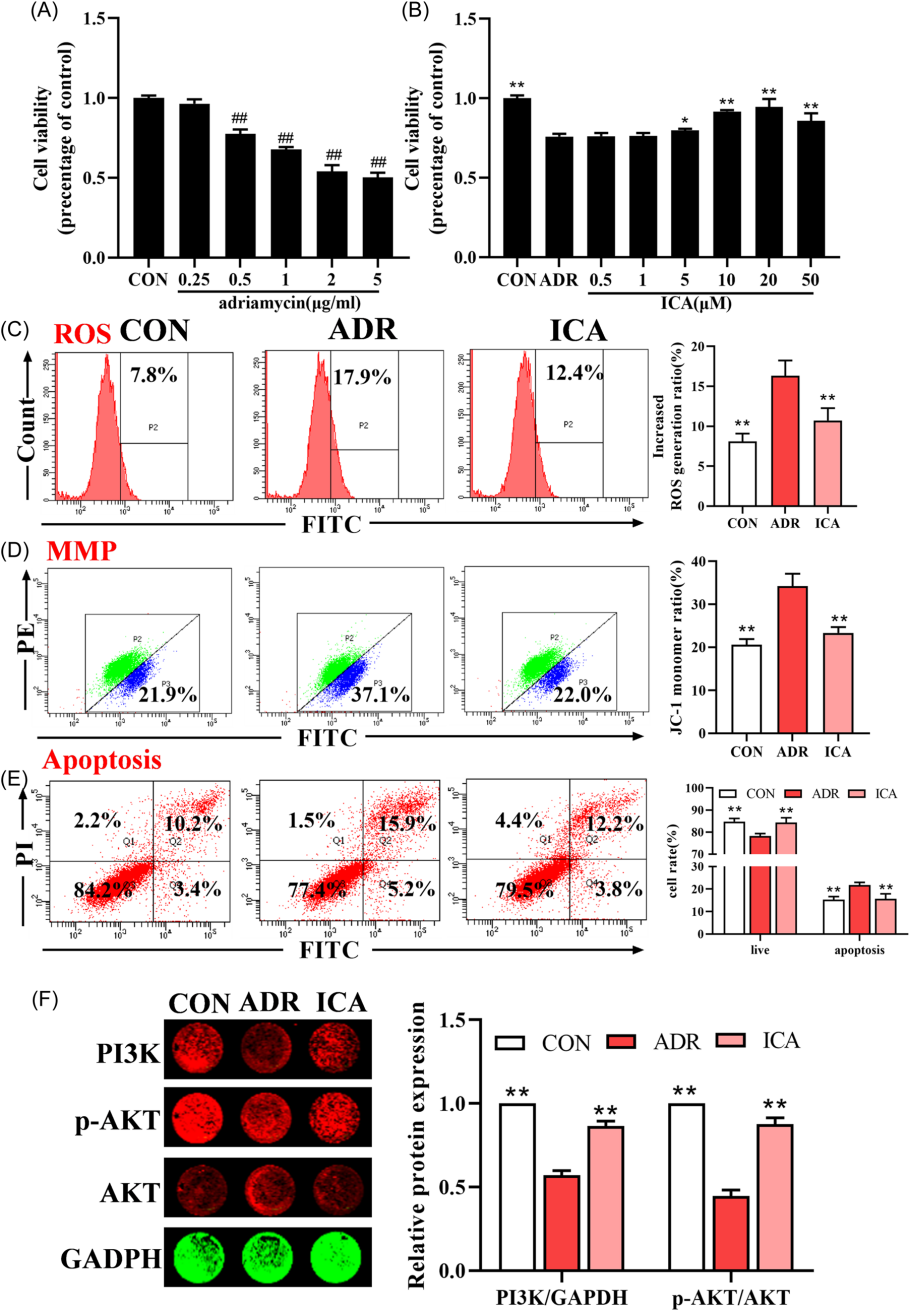
Effects of Icariin on mitochondrial apoptosis in adriamycin‐induced NRK‐52e cells. CON: control. (A) Effects of adriamycin (0.25, 0.5, 1, 2, and 5 μg/mL) on the viability of NRK‐52e cells. (B) Effects of icariin (0.5, 1, 5, 10, 20, and 50 μM) on the viability of adriamycin (0.5 μg/mL)‐induced NRK‐52e cells. (C) The level of ROS in NRK‐52e cells was measured by flow cytometry. (D) The level of MMP in NRK‐52e cells was measured by flow cytometry. (E) The level of apoptosis in NRK‐52e cells was measured by flow cytometry. (F) Western blot detection of PI3K/AKT signaling pathway‐related protein levels. Data are expressed as the mean ± *SD*, *n* = 3 rats per group, *p < .05, **p < .01 compared with the ADR group. ADR, adriamycin‐induced nephropathy; EPI‐H, Epimedium sagittatum Maxim high dose group; EPI‐L, Epimedium sagittatum Maxim low dose; ICA, Icariin; ROS, reactive oxygen species.

**Figure 10:**
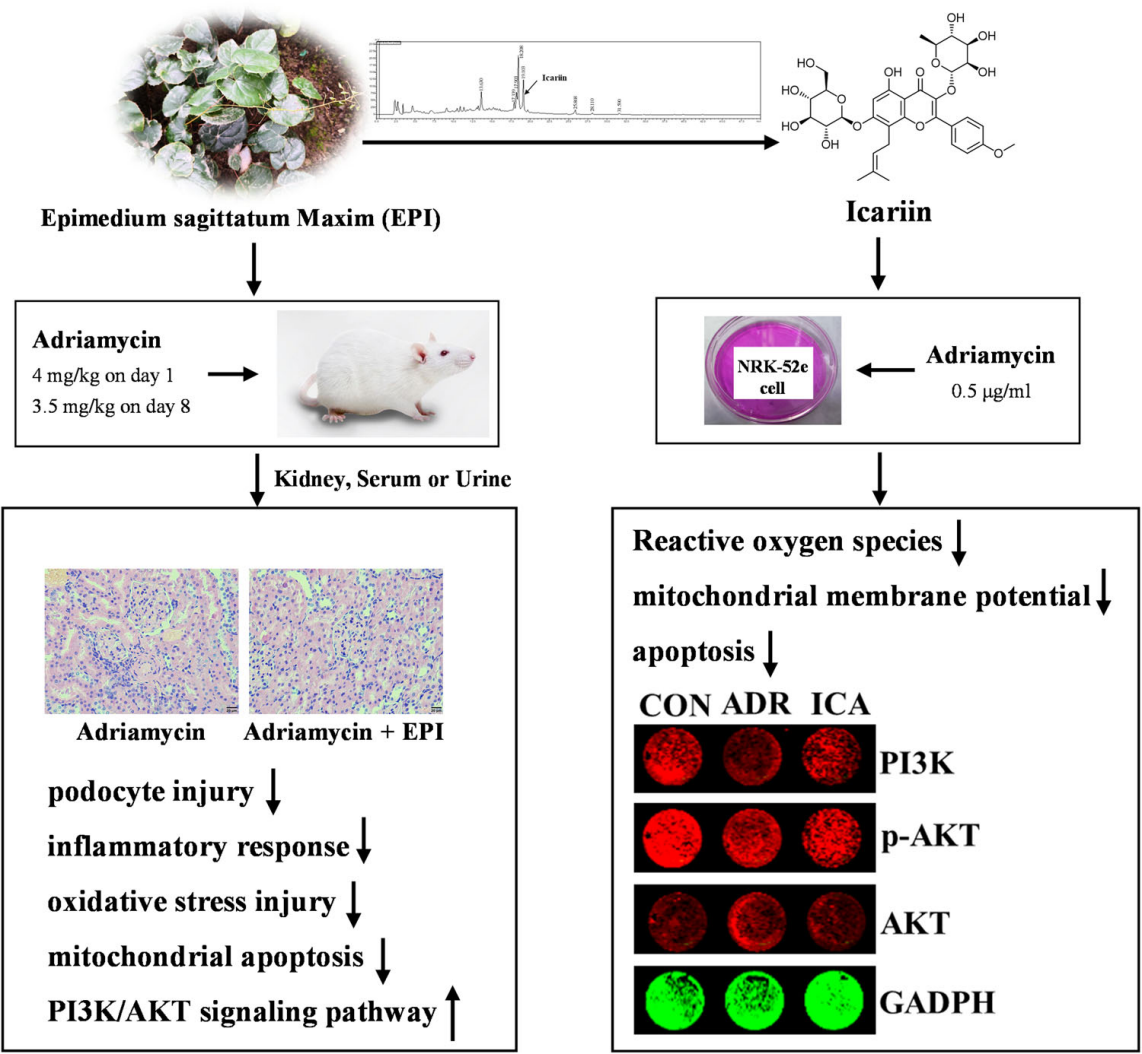
Mechanism of study on the intervention effect of Epimedium sagittatum Maxim (EPI) on adriamycin nephropathy in rats. EPI can improve podocyte injury, reduce inflammatory response, improve oxidative stress injury and inhibit apoptosis in rats with adriamycin nephropathy, and its mechanism may be related to PI3K/AKT signaling pathway, icariin may be the pharmacodynamic substance responsible for this effect.

The study found that icariin is the representative component of EPI, and the 2020 edition of Chinese Pharmacopoeia stipulated that icariin is the quality evaluation standard of EPI.^18^ Our results suggest that adriamycin significantly reduced NRK‐52e cell viability in a dose‐dependent manner and icariin can significantly increase cell viability of adriamycin‐induced NRK‐52e cells in a dose‐dependent manner (Figure 9A,B). In addition, icariin reduced the levels of ROS, JC‐1, and apoptosis in adriamycin‐induced NRK‐52e cells (Figure 9C–E). Icariin could also significantly increase the protein levels of PI3K and p‐AKT in adriamycin‐induced NRK‐52e cells (Figures 9F and 10).

## 4 DISCUSSION

Adriamycin is widely used to treat solid tumors and hematologic malignancies; however, approximately 60% of patients develop nephrotoxicity, which greatly increases morbidity and mortality rates.^19^, ^20^ The basic process of adriamycin‐induced nephropathy is not well understood, but some researchers have suggested that it mainly involves oxidative stress injury, inflammatory response, and podocyte injury.^5^, ^7^ PA is a glucocorticoid that can be used to treat nephropathy in the clinic; however, PA has side effects, including dependence and allergic reactions. Medicinal plants from natural sources are associated with limited adverse reactions, providing important new clues for treating various chronic diseases.^21^ In this study, EPI ameliorated adriamycin‐induced kidney injury by inhibiting inflammation and mitochondrial apoptosis through the PI3K/AKT signaling pathway.

Renal pathological injury and dysfunction are the common features of adriamycin‐induced nephropathy, including glomerular injury and elevated levels of Cr, BUN, and urinary protein.^22^, ^23^ Our results showed that PA and EPI alleviated renal tubular epithelial cell injury and kidney interstitial inflammatory infiltration and reduced Cr, BUN, KIM‐1 MAU, and UP levels in rats with adriamycin‐induced nephropathy; EPI‐L and EPI‐H had the same improvement effect. In addition, podocyte injury is one of the characteristics of adriamycin‐induced nephropathy.^24^ Desmin expression is significantly correlated with podocyte injury,^7^ and nephrin is essential for podocytes to maintain the glomerular filtration barrier.^25^ The ultrastructure of the kidney tissue showed that PA and EPI could improve the damage to the glomerular basal structure and foot process fusion; PA and EPI could also downregulate desmin and upregulate nephrin in adriamycin‐induced nephropathy rats. EPI‐H had a better effect overall, suggesting that EPI could alleviate podocyte injury caused by adriamycin.

Network pharmacology is of great significance for predicting the targets of pharmacological action in diseases and the possible signaling pathways involved.^26^ Through network pharmacology, we found that AKT1, TNF, and IL‐6 may be the core genes associated with EPI in the treatment of adriamycin‐induced nephropathy, and the PI3K/AKT signaling pathway is the potential key signaling pathway. The PI3K/AKT signaling pathway is involved in a variety of cellular functions including cell growth, differentiation, and apoptosis.^27^ AKT signaling can reduce cellular oxidative stress, inhibit the release of proinflammatory cytokines, and ameliorate podocyte injury.^17^ Our results showed that EPI could increase the levels of PI3K and p‐AKT in rats with adriamycin‐induced nephropathy. Activation of AKT inhibited phosphorylation of κB kinase at Thr23, which is involved in regulating inflammation.^28^ Inflammation plays a key role in the progression of adriamycin‐induced nephropathy and is thought to be an important cause of podocyte damage.^22^ The inflammatory marker MCP‐1 can recruit macrophages to kidney tissue,^29^ macrophages can release inflammatory mediators including TNF‐α and IL‐1β and the NF‐κB signaling pathway can be activated, which in turn releases inflammatory factors.^30^, ^31^ Our results showed that the levels of IL‐1β, TNF‐α, TGF‐β1, IL‐6, MCP‐1, IL‐1β, and NF‐κB p‐p65 were decreased, and those of IL‐10 were increased, after PA and EPI treatment. EPI‐L and EPI‐H demonstrated the same treatment effect, suggesting that EPI could inhibit inflammatory response in adriamycin‐induced nephropathy rats.

Studies have revealed that exposure to adriamycin in rats can lead to excessive ROS and cause oxidative stress in the kidney.^32^ SOD and GSH‐Px can effectively remove ROS and terminate the free radical reaction, and MDA can reflect the degree of peroxidation damage.^33^ In addition, it has been reported that the PI3K/AKT pathway can activate the Nrf2 pathway, further promote the expression of HO‐1 and NQO‐1, and upregulate various cellular antioxidant enzymes to protect tissues from oxidative stress.^34^, ^35^, ^36^ In this study, PA and EPI reduced ROS and MDA levels and increased GSH‐Px, T‐SOD, Nrf2, HO‐1, and NQO‐1 levels, suggesting that EPI can inhibit the oxidative stress response in adriamycin‐induced nephropathy.

Excessive ROS after kidney injury mediated by adriamycin plays a key role in activating the intrinsic apoptotic pathway through mitochondrial instability.^37^ Excessive ROS leads to Ca^2+^ influx, which in turn ROS production, reduces the MMP and promotes apoptosis.^28^, ^38^, ^39^ Chen et al.^40^ found that Danshen injection could improve adriamycin‐induced nephropathy by reducing apoptosis through PI3K/AKT signaling. In our study, PA and EPI decreased the levels of Ca^2+^ and apoptosis and increased MMP levels in primary renal cells, upregulated Bcl‐2, and downregulated Bax and Cleaved caspase‐3. The effects of EPI‐H were better overall. These results suggest that EPI ameliorates the symptoms of adriamycin‐induced nephropathy in rats by inhibiting mitochondrial apoptosis. Icariin is the main bioactive component of Epimedium, which has anti‐inflammatory, antioxidant, and immunomodulatory effects.^13^, ^41^ In addition, according to the 2020 edition of Chinese Pharmacopoeia, icariin is an indicator for the quality evaluation of EPI.^18^ The results showed that icariin could reduce ROS, MMP, and apoptosis levels and increase PI3K and p‐AKT levels in adriamycin‐induced NRK‐52e cells, suggesting that icariin may be the active ingredient of EPI in the treatment of adriamycin‐induced nephropathy.

## 5 CONCLUSION

In conclusion, EPI could ameliorate adriamycin‐induced nephropathy by reducing inflammation and apoptosis via the PI3K/AKT signaling pathway; icariin may be the pharmacodynamic substance responsible for this effect.

## AUTHOR CONTRIBUTIONS

**Ru Wang**: conceptualization; methodology; resources; software; writing—review & editing. **Mengnan Zeng**: conceptualization; methodology; software. **Beibei Zhang**: formal analysis; investigation; validation. **Qinqin Zhang**: formal analysis; investigation; validation. **Shuangshuang Xie**: formal analysis; investigation; validation. **Yingbo Hu**: data curation; visualization. **Ruyi Fan**: data curation; visualization. **Mengya Wang**: data curation; visualization. **Xiao Yu**: data curation; visualization. **Yuhan Zhang**: data curation; visualization. **Xiaoke Zheng**: conceptualization; methodology; software. **Weisheng Feng**: funding acquisition; project administration; supervision.

## CONFLICT OF INTEREST STATEMENT

The authors declare no conflicts of interest.

## ETHICS STATEMENT

All animal experiments have been approved by the Animal Ethics Committee of the Henan University of Chinese Medicine, Zhengzhou, China. Approval No. DWLL2018080003.

## Data Availability

The data of this study are available from the corresponding author upon reasonable request.
